# Reactive Oxygen Species in Venous Thrombosis

**DOI:** 10.3390/ijms21061918

**Published:** 2020-03-11

**Authors:** Clemens Gutmann, Richard Siow, Adam M. Gwozdz, Prakash Saha, Alberto Smith

**Affiliations:** 1 King’s British Heart Foundation Centre, King’s College London, 125 Coldharbour Lane, London SE5 9NU, UK; clemens.gutmann@kcl.ac.uk; 2 Vascular Biology & Inflammation Section, School of Cardiovascular Medicine & Sciences, British Heart Foundation of Research Excellence, King’s College London, SE1 9NH, UK; richard.siow@kcl.ac.uk; 3 Academic Department of Surgery, School of Cardiovascular Medicine & Sciences, British Heart Foundation of Research Excellence, King’s College London, London SE1 7EH, UK; adam.gwozdz@kcl.ac.uk (A.M.G.); Prakash.saha@kcl.ac.uk (P.S.)

**Keywords:** DVT, venous thrombosis, reactive oxygen species, oxidative stress, redox regulation, anticoagulation, thrombus resolution, dietary antioxidants, antioxidant defenses

## Abstract

Reactive oxygen species (ROS) have physiological roles as second messengers, but can also exert detrimental modifications on DNA, proteins and lipids if resulting from enhanced generation or reduced antioxidant defense (oxidative stress). Venous thrombus (DVT) formation and resolution are influenced by ROS through modulation of the coagulation, fibrinolysis, proteolysis and the complement system, as well as the regulation of effector cells such as platelets, endothelial cells, erythrocytes, neutrophils, mast cells, monocytes and fibroblasts. Many conditions that carry an elevated risk of venous thrombosis, such as the Antiphospholipid Syndrome, have alterations in their redox homeostasis. Dietary and pharmacological antioxidants can modulate several important processes involved in DVT formation, but their overall effect is unknown and there are no recommendations regarding their use. The development of novel antioxidant treatments that aim to abrogate the formation of DVT or promote its resolution will depend on the identification of targets that enable ROS modulation confined to their site of interest in order to prevent off-target effects on physiological redox mechanisms. Subgroups of patients with increased systemic oxidative stress might benefit from unspecific antioxidant treatment, but more clinical studies are needed to bring clarity to this issue.

## 1. Introduction

Deep vein thrombosis (DVT) accounts for around 10 million cases of thrombosis per year and is the third most prevalent cardiovascular disease (CVD) after myocardial infarction and stroke. The incidence of DVT increases with age and its complications are ranked among the top causes of death and poor quality of life in western countries [1]. As a complication of DVT, pulmonary embolism is the main driver of DVT-associated mortality. Venous reflux in DVT-damaged veins (post-thrombotic syndrome) is, however, the main driver of DVT-associated morbidity, causing pain, swelling and, when severe, chronic skin ulceration. DVT is therefore a major contributor to the global disease burden [1]. 

Anticoagulation is the most common approach taken when treating DVT, but although these drugs inhibit thrombus propagation, they are associated with an increased risk of pathological bleeding such as stroke. Anticoagulants also have little effect on resolving existing thrombi, which occurs over time through a natural process of organization. Although enhanced natural thrombus resolution is associated with improved long-term outcome [2], interventional (endovascular or open surgery) approaches of thrombus resolution are not always effective and are associated with side effects that limit their use in the majority of patients [3]. The development of novel treatment modalities, which block thrombus formation and/or enhance thrombus resolution but, at the same time, carry fewer side effects, are therefore desirable and will only come from an improved understanding of the regulatory mechanisms involved in these processes. 

**Figure 1:**
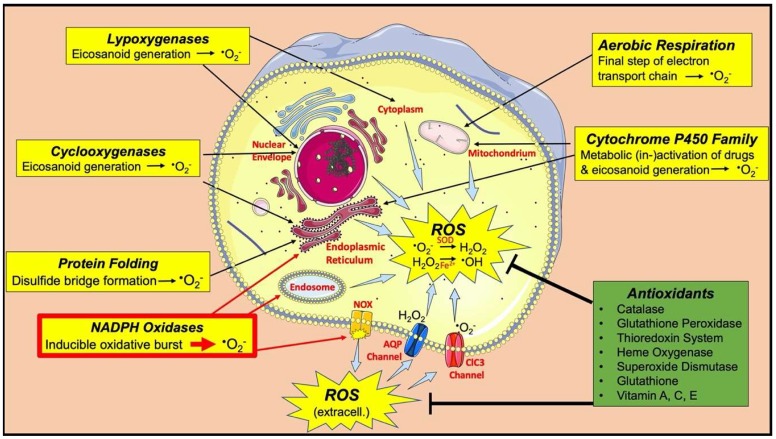
Major sources of reactive oxygen species (ROS) and antioxidant defense mechanisms. NADPH oxidases (NOX; highlighted in red) appear to be the most important source of ROS involved in processes related to the formation and resolution of venous thrombi.

As discussed in this review, numerous studies have shown that both thrombus formation and its resolution may be regulated by reactive oxygen species (ROS). Certain intracellular ROS, such as superoxide (O_2_^−^) and hydrogen peroxide (H_2_O_2_), have physiological functions as second messengers in signalling pathways, while pathologic generation or decreased antioxidant scavenging of extra- and intracellular ROS (oxidative stress) lead to damage of proteins, lipids and DNA, resulting in modified functions of these molecules as well as dose-dependent programmed or unprogrammed cell death [4]. ROS are generated as by-products of aerobic respiration within mitochondria [5], during oxidative folding of proteins (endoplasmic reticulum [6]), as well as enzymes of the cytochrome p450 (endoplasmic reticulum and mitochondria [7]), cyclooxygenase (endoplasmic reticulum, nuclear envelope [8]), lipoxygenase (nuclear envelope, cytoplasm [9]) and NADPH oxidase (NOX) family (endoplasmic reticulum, endosomal and plasma membranes [10], Figure 1) [4]. NOX enzymes appear to be the most important source for ROS involved in processes related to DVT. Depending on isotype and cell type, these membrane-bound enzymes release O_2_^−^ into intra- or extracellular compartments [10]. Superoxide may then react (spontaneously or catalysed by superoxide dismutase) to form H_2_O_2_. If H_2_O_2_ molecules encounter transition metals such as Fe^2+^ ions, they can be converted into highly reactive hydroxyl radicals (**•**OH, Fenton Reaction [11]), leading to oxidative damage [4]. Several antioxidant enzymes (superoxide dismutase [12], catalase [13], glutathione peroxidases [14], heme oxygenase [15], thioredoxin system [16]) and small molecule antioxidants (glutathione [17], vitamin A, C and E [18]) are produced to scavenge ROS, thereby limiting these detrimental effects [18]. These redox mechanisms are essential for normal physiological homeostasis, but their dysregulation may have pathological consequences. In this review, we provide an overview of how ROS and perturbations in redox homeostasis may be involved in the mechanisms that give rise to the formation and resolution of venous thrombi. We also aim to highlight the clinical potential of targeting these processes. 

## 2. Venous Thrombus Formation

**Figure 2:**
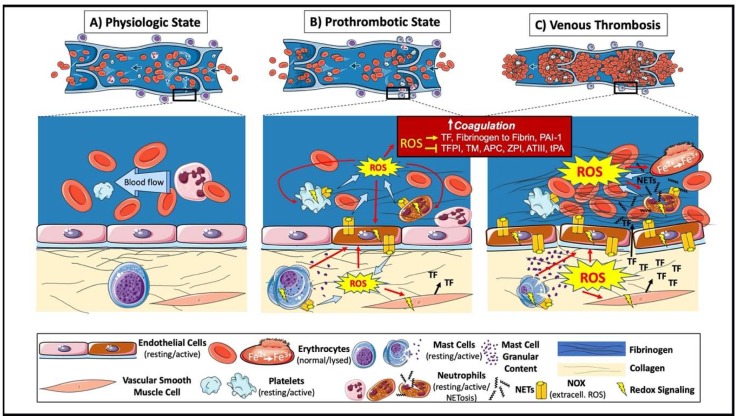
ROS in the formation of a venous thrombus. (**A**) Physiologic venous circulation. (**B**) At sites of blood stasis, activated endothelial cells increase ROS luminally (contributing directly and indirectly to recruitment and activation of leukocytes and platelets) and abluminally (contributing directly and indirectly to the activation of mast cells and vascular smooth muscle cells). Activated cells contribute to further ROS generation and use redox signaling to exert their prothrombotic effects, including activation of the coagulation system and platelet aggregation; mast cell degranulation and tissue factor (TF) production within the vein wall. (**C**) Increased coagulability of blood in the presence of low flow (two of Virchow’s Triad) provides the environment for venous thrombus formation. In the forming thrombus, erythrocytes (major component) and platelets are entrapped by large amounts of cross-linked fibrin. The oxidative stress leads to oxidation of hemoglobin (Fe^2+^) to methemoglobin (Fe^3+^), resulting in a positive feedback on oxidative stress and thrombus formation. Neutrophil extracellular traps (NETs) provide an additional scaffold and contribute to further oxidative stress and thrombus formation.

The development of a venous thrombus requires activation of the endothelium, platelets and sterile inflammation (involving neutrophils, mast cells and monocytes), stimulated by the interaction between at least two of Virchow’s triad of factors: reduced blood flow; endothelial disturbance; and the hypercoagulability of blood. This process leads to the activation of the coagulation cascade with the generation of large amounts of thrombin, converting fibrinogen to fibrin. Venous thrombi have a laminar structure consisting of layers of platelets, leukocytes and fibrin (‘lines of Zahn’) that encompass the main erythrocyte mass that is very different from the structure of arterial, platelet-rich, “white” thrombi and the amorphous clots of coagulated blood [19]. Leukocytes (neutrophils) can be found attached to the endothelium at the sites of thrombus formation, usually at sites of reduced blood flow such as vein valve pockets and dilated sinuses [20,21]. Activated neutrophils can release neutrophil extracellular traps (NETs), consisting of decondensed chromatin and DNA [22]. These NETs can enhance coagulation through the direct induction of platelet aggregation, erythrocyte recruitment and fibrin deposition [22]); as well as causing endothelial activation and damage [23]. The disrupted and activated endothelial barrier can expose or release prothrombotic proteins, such as collagen, tissue factor, von Willebrand Factor [24,25], and chemotactic proteins, such as cytokines and surface adhesion molecules [26], into the blood, that support further coagulation, platelet aggregation and leukocyte recruitment. Mast cells residing in the vein wall also contribute to venous thrombus formation through endothelial activation and Weibel–Palade body release [27], which, at least in part, is mediated by histamine [28]. The modulation of many of these contributing factors can inhibit thrombus formation. Venous thrombus formation therefore depends on more than just activation of the coagulation cascade, and processes involved in DVT formation other than the coagulation cascade might hold potential for novel treatment options. Identifying pharmacological targets will, however, require an improved understanding of the molecular mechanisms involved in thrombus formation. ROS appear to be important regulators of various processes involved in this phenomenon and the following sections will explore their involvement in more detail (Figure 2).

## 3. ROS and Coagulation

### 3.1. ROS in Signaling Pathways Modulating Procoagulant Responses 

ROS stimulates coagulation by increasing the expression of tissue factor (TF) in endothelial cells [29], monocytes [30] and vascular smooth muscle cells (VSMCs) [31], with ROS-generating NOX enzymes appearing to be important contributors [30,31,32]. The stimulation of protease-activated receptors (PARs) may also lead to endothelial TF induction via mitochondrial ROS signaling [33]. The coagulation system can also induce the generation of ROS with thrombin, inducing a biphasic ROS increase in endothelial cells, in which the second peak is attributed to transcriptional upregulation of the NOX subunit p22phox via p38 MAPK and PI3K/Akt [34]. 

### 3.2. ROS Oxidative Modification of Proteins That Regulate Coagulation 

ROS may favor a procoagulant state through oxidative modifications of proteins involved in coagulation. Tissue factor pathway inhibitor (TFPI), the only physiologic regulator of TF activity, can be inhibited by oxidative stress and exert a procoagulant effect [35]. ROS can also directly inactivate major anticoagulant proteins, protein C (PC) [36] and its upstream agonist thrombomodulin (TM) [37]. Larger venous thrombi form more rapidly in *SOD^-/-^* mice, which is thought to be caused by reduced SOD1-mediated protein C activation and SOD1-mediated protection of TM [38]. ROS can exert a prothrombotic role by oxidizing fibrinogen, which is then more readily converted to fibrin [39], and reduce the interaction between thrombin and anticoagulants such as protein C, the antithrombin III-heparin complex, and TM [40]. The heparin-binding capability of antithrombin is also reduced following oxidation by hydrogen peroxide (H_2_O_2_) [41] or lipid peroxides [42]. Oxidized phospholipids suppress the anticoagulant function of the serpin, protein Z-dependent protease inhibitor (ZPI), a specific inhibitor of membrane-associated factor Xa (FXa) that requires protein Z, phospholipid, and calcium as cofactors [43]. Eosinophils have been shown to propagate coagulation, hemostasis and thrombotic disease through 12/15-lipoxygenase-derived oxidized phospholipids [44].

## 4. ROS and Platelets

There is increasing evidence that platelets have a role in the formation of venous thrombi [45], and changes in platelet reactivity affect the risk of DVT [46]. Platelet function is regulated by ROS and impairments in these processes might be responsible for adverse outcomes in patients at risk of developing a DVT. 

ROS affects the expression of P-selectin, the circulating levels of which are associated with an increased risk of venous thromboembolism (VTE) [47,48], which may result from its interaction with PSGL1 on neutrophils [49]. The expression of P-selectin [50] and CD40L [51] that are transferred to the platelet surface upon activation is, in part, ROS-dependent. Platelet NOX2 is upregulated and plasma levels of soluble P-selectin and soluble CD40L (sCD40L)are elevated in obese patients who have elevated oxidative stress and an increased risk of DVT [52], while the plasma levels of these proteins are decreased in women with hereditary deficiency of NOX2 [53]. Increased levels of soluble CD40L can enhance platelet activation, aggregation, platelet-leukocyte conjugation and further ROS production [54]. 

Intraplatelet ROS activates platelets by oxidizing arachidonic acid, thereby generating isoprostanes [55]. Patients with hypercholesterolemia [56], diabetes mellitus [57], homozygous homocystinuria [58] and women with obesity [59], have increased circulating levels of isoprostanes associated with persistent platelet activation. Each of these conditions carries an increased risk of DVT [60,61]. ROS may also indirectly enhance platelet reactivity by negatively affecting endogenous mechanisms involved in platelet inhibition, such as the scavenging of nitric oxide (NO) that is synthesized by endothelial cells and exerts an anti-platelet aggregating effect [62]. 

The importance of ROS-mediated effects on platelet activation is evidenced by the diminished activation seen in the presence of antioxidants such as catalase [55], N-acetylcysteine (NAC) [63], polyphenols [64], vitamin C [64], and vitamin E [65], and the increased activation seen in the presence of ROS donors [55]. Knockout of the antioxidant enzyme glutathione peroxidase-3 (GPX-3) results in increased platelet-dependent thrombosis in mice [66], whereas mice overexpressing the GPX-1 isoform were protected from platelet hyperactivity and age-dependent increased susceptibility to experimental venous thrombosis after ligation of the inferior vena cava (IVC) [67].

The essential involvement of NOX enzymes in platelet reactivity is evident from the impaired platelet activation in patients with X-linked chronic granulomatous disease (CGD), characterized by a lack of the NOX subunit gp91phox (NOX2) [55]. Similar observations have been obtained by experimental genetic or pharmacological inhibition of NOX enzymes [55]. Ex vivo investigation of NOX subtype involvement in platelet ROS production and platelet activation revealed that treatment with collagen-related peptide, a GPVI-specific agonist, resulted in NOX1, but not NOX2-dependent ROS production and prothrombotic thromboxane A2 production. NOX1-dependent and NOX2-independent ROS production were also seen upon exposure of platelets to collagen, and ex vivo thrombus formation is impaired in platelets from NOX2 knockout mice [68]. These results hint at a currently unknown ROS-independent function of NOX2 in platelets, although this may be more plausibly explained by the effects of NOX2 knockout in leukocytes and/or endothelial cells, which could have contributed to thrombus formation and platelet reactivity [68]. In vivo studies show that NOX2 knocked out in both hematopoietic and endothelial cells is crucial for neutrophil-platelet interactions during TNF-α-induced venous inflammation that is coupled to thrombosis [50]. Impaired microvascular perfusion in NOX2-deficient mice, occurring through activated coagulation and/or P-selectin-mediated platelet adhesion, is rescued by antioxidant treatment using ascorbate [69]. GPVI stimulation on mouse NOX2-knockout platelets results in reduced ROS generation and platelet activation, while NOX1 knockout platelets showed only selective defects in G-protein–coupled receptor–mediated platelet activation induced by thrombin [70]. Activation of class III phosphoinositide 3-kinase (PI3K) which is important for thrombosis, but dispensable for hemostasis, is also dependent on NOX assembly and subsequent ROS generation [71].

## 5. ROS and Extracellular Traps

The formation of a venous thrombus is particularly dependent on sterile inflammation. One inflammatory mechanism, the release of extracellular traps (NETs), comprised of decondensed chromatin and DNA from activated neutrophils, that was originally thought to be limited to host defense in infection, is also of particular importance to DVT formation [72]. The prothrombotic effect of NETs were initially revealed in experimental models, both in vitro and in vivo [22]. Subsequent studies have confirmed the pathophysiological role of NETs in venous thrombosis, demonstrating their influence on the clotting cascade and identifying essential proteins involved in NETosis [73]. Although extracellular trap formation (ETosis) has mainly been investigated in neutrophils, other immune cells, such as mast cells, eosinophils, and macrophages, have more recently also been shown to release these structures [74]. A role for these sources of ETs in the initiation of DVT remains to be established.

Most pathways resulting in ET release involve signaling by ROS, generated by the phagocyte NADPH homologue, NOX2, which is present in both plasma and phagosome membranes [75]. Many NOX2 activators such as proinflammatory cytokines, lipopolysaccharide (LPS), toll-like receptor (TLR) agonists and chemical agents, such as phorbol 12-myristate 13-acetate (PMA), also trigger NET formation. Neutrophil NOX2 also regulates the function of surface receptors required for platelet–neutrophil interactions under thromboinflammatory conditions [50] and this may indirectly favor NETosis, since platelet–neutrophil interactions can induce NETosis through the binding of a P-selectin to its receptor, PSGL-1 [49]. 

A mechanistic explanation of how ROS enables the initial steps of NETosis, involving intracellular membrane disintegration followed by enzymatic chromatin decondensation, is the ROS-dependent translocation of neutrophil elastase (NE) and myeloperoxidase (MPO) from cytoplasmic azurophilic granules to the nucleus. NE cleaves nucleosomal histones and promotes chromatin decondensation, whereas MPO aids in chromatin decondensation during later stages [76]. Chromatin decondensation is also facilitated by ROS through PAD4-dependent citrullination [77]. The relevance of the ROS-dependent regulation of these processes for DVT formation has been shown in mouse models, with the finding that PAD4-inhibition abrogates NETosis [77], and that <10% of *PAD4^-/-^* mice develop venous thrombi 48hr after IVC ligation, compared with 90% of wild-type mice [78].

Taken together, the evidence suggests that signaling involved in NETosis requires ROS as second messengers, but also that NETosis may deploy ROS to mediate its extracellular functions. For example, MPO, an ROS-generating enzyme, is among the molecules extruded with NETs. Contrary to this, however, a recent study shows that MPO inhibits phospholipid-dependent coagulation [79], while acquired neutrophil MPO deficiency in man is associated with thrombotic disease [80]. These data suggest a regulatory function on blood coagulation beyond the known oxidative properties of MPO. The contribution of redox signaling in neutrophils to the formation of venous thrombosis would seem appropriate, given the extensive evidence for neutrophil involvement in this pathology. Experimental DVT induction in a conditional NOX2 knockout model would be an example of how this might be achieved. 

## 6. ROS and Erythrocytes

Erythrocytes are the main cellular constituent of venous thrombi. Although they are not thought to be major active contributors to DVT formation, some studies suggest that they contribute in a more passive way via oxidative mechanisms. Erythrocytes are enriched in iron as they contain a high amount of the oxygen-transport protein hemoglobin. Increased oxidative stress, as encountered at the sites of venous thrombi, is able to oxidize Fe^2+^ containing hemoglobin (Hb) to Fe^3+^-containing methemoglobin. The release of Fe^3+^ triggers a chain reaction, resulting in further erythrocyte lysis, oxidative stress and thrombus formation [81]. Hemoglobin also upregulates the expression of functional TF in macrophages and desensitizes TF to the effects of antioxidants [82]. Endogenous antioxidant enzymes such as hemeoxygenase-1 (HO-1) counteract this process. This is evident from experimental models that show that HO-1 knockout mice have increased thrombus size at the later stages of thrombus formation [83], while there is a higher risk of recurrent VTE in humans with variations in the HO-1 gene that reduce HO-1 activity [84]. Extracellular heme derived from lysed erythrocytes can also induce NETosis [85], as well as the NLRP3 inflammasome in macrophages via NOX2 [86], both of which may potentiate venous thrombosis [87].

Erythrocytes therefore appear to use oxidative mechanisms to passively promote DVT formation once the process has already started and they are entrapped within the growing thrombus. The degree to which this process is relevant remains unclear, however, as studies suggesting this mechanism were based on in vitro findings [82], performed under artificial oxidative stress in vivo [81] or in sickle cell mice with increased hemolysis [85], or were unable to rule out effects mediated by other cell types that accumulate in the thrombus [83,84].

## 7. ROS and Mast Cells

Mast cells (MCs) represent another sterile inflammation mediator that belongs to the innate immune system and has granules that contain both proinflammatory cytokines such as TNF-α and histamine, as well as antithrombotic factors, such as tissue plasminogen activator (tPA) and heparin [88]. MCs can be found in the wall of blood vessels and are activated and degranulated in response to the hypoxia that may follow the stagnation of blood flow in veins [89]. The numbers of MCs close to the sites of venous thrombus formation are reduced, and when depleted prior to thrombus induction, thrombosis is abolished, leading to the suggestion that there is a link between MCs and venous thrombogenesis [27]. A putative mechanism linking MCs with thrombosis involves MC histamine-induced activation of vWF release from endothelial Weibel–Palade bodies, but it is still unclear as to how hypoxia induces activation of MCs [27]. It must be noted, however, that vein wall MC numbers are reported to be increased in DVT patients and express a profibrinolytic phenotype [88].

It is generally accepted that MCs generate ROS and can be activated by ROS [90,91]. The inhibition of MC activation downregulates ROS production, while antioxidants prevent MC degranulation. H_2_O_2_ mediates the increased susceptibility to DVT in aged mice, whereas overexpression of glutathione peroxidase-1 is protective in mice [67]. NOX2 is a major source of ROS in MCs [92] and ROS activate redox-sensitive calcium channels that facilitate the rise in cytoplasmic calcium needed for MC degranulation [93].

## 8. ROS and the Complement System

The complement system exerts its effects through an enzymatic cascade similar to the coagulation cascade, with many interactions between these two processes that confer prothrombotic capabilities to this system. High levels of the C3 fragment are associated with a high risk of DVT in man [94], while thrombus weight strongly correlates with C5a levels in experimental DVT [95]. Although the complement system can increase extracellular ROS through the activation of neutrophils [96], the predominant inflammatory cell type present in the forming thrombus, little work has been afforded to the involvement of ROS in complement-mediated DVT-promoting processes.

## 9. ROS and Calcium Homeostasis

Calcium is an essential component of the human diet and is required for the activity of many enzymes, including those involved in processes related to DVT. Many of these calcium-based signaling systems interact with redox signaling pathways. These interactions are bidirectional, with calcium channels on plasma and organellar membranes regulated by oxidative modifications [97,98,99,100], and with ROS generators such as NOX enzymes regulated by calcium [101,102,103]. Although this interplay has been documented in many different cell types and a similar regulation is likely to take place in DVT, it has to be noted that studies in DVT models are lacking. 

Platelet activation, for instance, relies on cytoplasmic calcium surges. These calcium surges occur mainly through store-operated calcium entry (SOCE), where calcium is first released from intracellular stores, followed by entry through the plasma membrane. In SOCE, the initial calcium release from intracellular stores is mediated by the stromal interaction molecule 1 (STIM1) protein, while the following calcium influx from the extracellular space is mediated by the plasma membrane channel Orai1 [104]. Both of these key proteins are under redox control. STIM1 disulfide formation decreases calcium influx [97], while S-glutathionylation of STIM1 leads to Orai1 activation, independent of calcium release from stores [98]. Another calcium channel through which calcium enters platelets from the extracellular space is the transient receptor potential channel 6 (TRPC6) [104]. Not observed in platelets but seen in kidney cells [99] as well as B lymphoma cells [100], TRPC6 was found to directly interact with plasma membrane NOX2, with ROS generated from this NOX2 exerting a positive feedback loop on TRPC6 activity. 

STIM1, Orai1 and TRPC6 are also involved in angiogenesis, a process important for thrombus resolution, as outlined later in this article [105]. 

Calcium–ROS interplay is also important for processes by which leukocytes might contribute to DVT formation. In neutrophils, for instance, NOX2 is essential for neutrophils’ role in DVT formation. Activation of this enzyme in neutrophils relies on a calcium signaling pathway [103]. In mast cells, ROS activates redox-sensitive calcium channels that facilitate the rise in cytoplasmic calcium needed for MC degranulation [93].

## 10. Venous Thrombus Resolution

It is well known that rapid natural thrombus resolution is associated with improved long-term outcome [2]. Current treatment options for rapid thrombus removal by thrombolysis with or without mechanical means have, however, significant hemorrhagic side effects and are therefore only deployed in severe cases in which immediate vessel recanalization is required [3]. A better understanding of the processes that enhance natural resolution might give rise to novel therapies that would especially benefit DVT patients with long-term complications such as recurrent VTE, post-thrombotic syndrome or chronic thromboembolic pulmonary hypertension (CTEPH).

**Figure 3:**
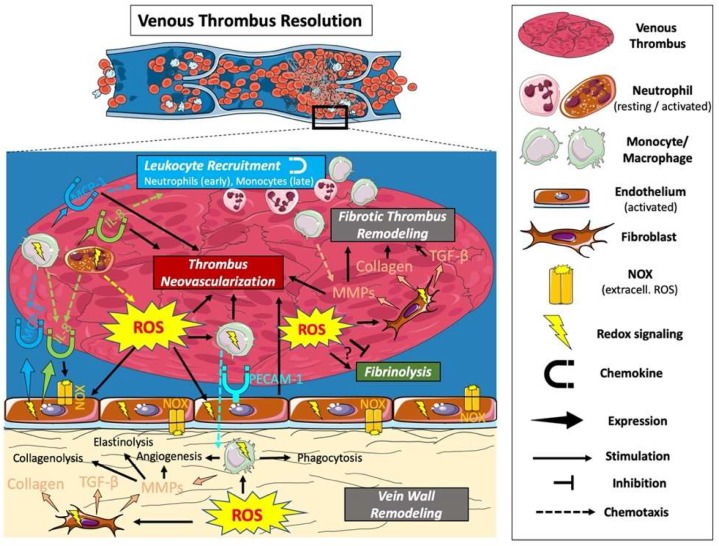
The role of ROS in the resolution of a venous thrombus. Oxidative stress at the site of a venous thrombus (intra- and abluminal) leads to the recruitment of leukocytes (with neutrophils predominating in the early, and monocytes predominating in the later phase of resolution) and fibroblasts. ROS-mediated cell recruitment results from direct chemotactic effects, or indirectly through increased expression of chemokines and adhesion molecules. The recruited cells promote thrombus resolution through phagocytic action removing red cell mass, thrombus and vein wall remodeling including fibrin degradation and the stimulation of new ECM deposition and neovessel formation. There are conflicting reports on whether fibrinolysis is stimulated or inhibited by ROS.

Venous Thrombus resolution is a process of tissue organization and remodeling that resembles the processes that take place during normal wound healing. It requires the concerted action of the following processes: fibrinolysis; proteolysis; inflammation; and angiogenesis. In the early stage of resolution, a gradual process of remodeling replaces the fragile erythrocyte- and fibrin-rich matrix of the thrombus with collagen and other extracellular matrix proteins [26]. Angiogenic stimuli, including the production of vascular endothelial growth factor within the thrombus, promote the formation of neovascular channels within the thrombus [19]. Leukocytes are important mediators of these processes, with neutrophils predominating in the early, and monocytes in the late, stages of thrombus resolution. Each of these processes can be modulated by ROS (Figure 3).

## 11. ROS and Fibrinolysis

Tissue-type and urokinase-type plasminogen activators (tPA and uPA) are found within mononuclear cells that accumulate in experimental venous thrombi [106,107]. It appears, however, that only uPA activity is the main determinant of thrombus resolution and that this is associated with the accumulation of cells derived from the bone marrow, possibly monocytes [108,109]. Deficiency in either of the two plasminogen activator inhibitors, PAI-1 and PAI-2, results in enhanced resolution [110].

ROS are intimately involved in the regulation of the fibrinolytic system. Early in vitro studies have shown that oxidation reduces the ability of fibrinogen to activate tPA [39] and that expression of PAI-1 in endothelial cells [111], VSMCs [112] and other cells [113] is dependent on ROS. PAI-1 inhibits uPA in addition to tPA and ROS-dependent downregulation of PAI-1, which could thereby influence the resolution process. NOX enzymes seem to be an important source of the ROS involved in this process [112,114]. The signaling processes involved in PAI-1 upregulation involves the redox-controlled transcription factor AP-1 upon oxidative stress and insulin stimulation [115], whereas hypoxia-inducible factor 1 (HIF-1) is involved upon thrombin stimulation [112]. In line with the ROS-dependent regulation of PAI-1, the antioxidant Vitamin E has been shown to improve fibrinolytic function by reducing PAI-1 activity in patients with coronary spastic angina, associated with a decrease in oxidative stress [116]. 

There are, however, conflicting reports showing that ROS are capable of stimulating fibrinolysis by inactivating PAI in vitro [117] and may prevent hypoxia-mediated PAI upregulation [118]. Later experimental in vivo studies also show that, while PAI expression can be upregulated by ROS in adipose tissues [119] and in hyperoxia-induced oxidative stress in the lung [120], PAI levels may be increased in hepatocytes in the face of reduced ROS activity caused by overexpression of the antioxidant enzyme GPX [121]. 

The fibrinolytic system can be modulated by ROS in a variety of ways. The conflicting observations regarding the direction of this modulation imply a complex regulation that is dependent on the cell type, stimulus and whether ROS mediate their effects as part of specific second messengers or as part of unspecific oxidative stress.

## 12. ROS and Leukocyte Recruitment

Venous thrombi are quickly invaded by circulating leukocytes, and animal models have shown that thrombus resolution is impaired when the early neutrophil [122] or late monocyte [108,123] invasion is restricted. Resolution, on the other hand, is enhanced when monocyte numbers are increased [123]. Leukocyte recruitment is mediated by a variety of processes, all of which can be modulated by ROS.

ROS can directly act as chemoattractants for neutrophils in vitro and in vivo [124,125] and neutrophil chemotaxis can be inhibited by increased circulating presence of the antioxidant enzyme TRX [126]. Cell culture supplementation with different ROS also results in increased leukocyte adhesion molecule expression and leukocyte endothelial adhesion [127]. Cell adhesion molecules (CAMs), in particular platelet-endothelial CAM-1 (PECAM-1), are important for leukocyte thrombus invasion. The thrombi of PECAM-1 knockout mice have an impaired resolution and contain fewer macrophages in an IVC ligation model [128]. PECAM-1 is also downregulated in plasma of patients with non-resolving DVT and within unresolved thrombi of chronic thromboembolic pulmonary hypertension (CTEPH) patients [128]. Oxidative stress induces transendothelial migration of monocytes via phosphorylation of PECAM-1 in vitro [129], while PECAM-1 also downregulates the antioxidant enzyme heme-oxygenase-1 (HO-1) via the master antioxidant transcription factor Nrf2 and modifies intracellular ROS levels in human endothelial cells [130]. 

IL-8 is a chemokine produced by endothelial cells, monocytes and neutrophils, and it recruits neutrophils and, to a lesser extent, monocytes. Increased circulating levels of IL-8 are associated with an increased risk of recurrent DVT [131], while increased levels are associated with increased thrombus resolution in an experimental in vivo model [132]. Patients with chronic granulomatous disease (CGD) have gene mutations that give rise to impaired NOX function. Neutrophils isolated from CGD patients produce 2- to 4-fold more IL-8 and show a sustained IL-8 mRNA response after fMLP stimulation compared with normal neutrophils, while normal neutrophils treated with an ROS scavenger or NOX inhibitor have similar IL-8 responses to CGD neutrophils. Taken together, these data suggest that NOX activity reduces neutrophil IL-8 production [133]. In line with this, exposure of mouse neutrophils to extracellular H_2_O_2_ potently inhibits nuclear translocation of NF-κB, which induces the expression of proinflammatory cytokines such as IL-8, suggesting that extracellular oxidative stress reduces IL-8 production [134]. Contrary to expectations, ROS scavengers were shown to decrease the production of IL-8 in LPS- [135] and IgE-mediated [136] human neutrophil activation. A recent study in human neutrophils hinted at a more complex involvement of NOX. While intracellular IL-8 levels of fMLP stimulated neutrophils are increased upon NOX inhibition, extracellular release of IL-8 is reduced under the same condition, suggesting interference in the release mechanism [137]. The conflicting evidence of ROS involvement in neutrophil IL-8 regulation may result from differences in and off-target effects of neutrophil activators and ROS inhibitors. The effects of ROS on IL-8 regulation also vary according to cell type. In macrophages, for example, extracellular oxidative stress has no effect on cytokine production [134], while in human umbilical vein endothelial cells (HUVECs), exogenous superoxide upregulates IL-8 mRNA expression [32]. Besides being regulated by ROS, IL-8 induces ROS production in endothelial cells via NOX1 [138]. To what degree ROS-mediated IL-8 chemotaxis is important to leukocyte recruitment in thrombus formation (detrimental) or leukocyte recruitment in thrombus resolution (beneficial) remains to be studied.

Monocyte chemotactic protein-1 (MCP-1) is expressed by various vascular cells such as endothelial cells, VSMCs, fibroblasts, monocytes and macrophages [139]. MCP-1 mediates monocyte recruitment into the thrombus and administration of MCP-1 accelerates the organization and resolution of venous thrombi [123,140]. Although the influence of ROS on MCP-1 regulation has yet to be studied in thrombosis models, functional MCP-1 studies hint at a possible redox regulation. MCP-1 expression in human endothelial cells stimulated by IL-4 reveals the involvement of intracellular ROS, most likely generated by NOX enzymes [141]. Similar results were obtained in retinal endothelial cells exposed to ischemia [142] and in hepatic stellate cells upon alcoholic liver injury [143]. Besides this potential involvement of ROS as messengers in intracellular ROS signaling, experiments with ox-LDL, a marker of extracellular oxidative stress, have shown the upregulation of MCP-1 in endothelial cells and VSMCs [144].

## 13. ROS and Thrombus Neovascularization

Angiogenic stimuli drive the formation of neovascular channels within the thrombus and vessel wall during resolution. The importance of this process for resolution is evident from studies that have shown that overexpression and inhibition of the most potent angiogenic factor, VEGF, enhances and impairs thrombus resolution, respectively [145,146]. It has become clear that thrombus neovascularization also depends on leukocyte recruitment, with monocytes being one of the most important effector cells in this process [147]. It is therefore not surprising that the molecules involved in leukocyte-attraction PECAM-1, IL-8, MCP-1 also have proangiogenic activity [128,132,145,148], all of which we have described as being modulated by ROS.

Within a newly formed venous thrombus, pO_2_ levels are only 10% of that in circulating venous blood. This relative hypoxia is associated with increased levels of HIF-1 and angiogenic factors within the thrombus [89]. Hypoxia is also a prime activator of ROS and there is extensive evidence for a proangiogenic role for extracellular oxidative stress and intracellular, NOX-dependent redox signaling [149]. The main mechanism of oxidative stress-induced angiogenesis involves HIF-1/VEGF signaling, but VEGF-independent mechanisms, such as the ones mediated by lipid oxidation products and the downstream TLR2/MyD88 pathway, have also been reported [149,150]. The extracellular chaperone glypican-1 protects VEGF from oxidative damage, highlighting the role of angiogenesis in oxidative environments [151]. Thrombin, for instance, was shown to activate the HIF-1 signaling pathway and to increase VEGF expression in VSMCs [112]. Thrombin-stimulated in vitro angiogenesis is diminished by NOX2 or NOX5 knockdown in endothelial cells [152]. Consistent with this, several antioxidants, such as NOX inhibitors, free radical scavengers and overexpression of antioxidant enzymes, inhibit angiogenesis [153,154,155].

## 14. ROS and Fibrotic Thrombus Remodeling

The purpose of thrombus resolution is to restore lumen patency and, hence, vessel function. The exacerbation or impairment of mechanisms within this ‘intravascular wound healing’ process can lead to chronic, fibrotic thrombi and vein walls, causing persistent impediment to blood flow and vessel dysfunction, respectively.

During the early resolution process, the fragile erythrocyte- and fibrin-rich thrombus is replaced by collagen and, depending on the balance between profibrotic signals and degradative protease activity, the thrombus and vein wall may become fibrotic, as evidenced by the association seen between impaired thrombus resolution and elevated collagen deposition [122,128]. Leukocytes, vascular smooth muscle cells (VSMCs), and myofibroblasts together with the expression of profibrotic factors such as TGF-β within the thrombus and the adjacent vein wall, are key drivers of the remodeling in these structures [147]. 

TGF-β1-mediated fibroblast activation and the subsequent fibrotic response was shown to depend on NOX4-derived ROS in the lung [156]. Consistent with this, NOX4 knockout mice have defects in TGF-β1 signaling in a lung fibrosis model [157]. The involvement of NOX enzymes in TGF-β1 signaling has furthermore been reported in human pulmonary artery smooth muscle cells [158], cardiac fibroblasts [159] and dermal fibroblasts [160]. In contrast to this, NOX4 deficiency did not affect myofibroblast formation but nonetheless decreased collagen deposition in a mouse model of wound repair [161]. Thrombus and vascular wall remodeling are of particular importance in the pulmonary arteries, as long-term complications of pulmonary embolisms (PE) such as CTEPH are associated with high morbidity and mortality. A recent in vivo study has shown that pulmonary arterial (PA) endothelial dysfunction, induced by NOX-derived ROS, is an early event upon repetitive PE with sustained elevation of PA pressure [162]. VSMC proliferation is an important component of vascular remodeling, particularly important for the long-term patency of vein grafts. PAR2 receptors, for instance, mediate coagulation-independent actions of activated factor X (FXa), such as the proliferation of VSMCs. The intracellular signaling pathway of this process has been shown to involve NOX1-derived ROS in vitro [163].

These profibrotic mechanisms are counteracted by processes that drive the degradation of the ECM. Elastinolysis is believed to occur early and to be responsible for increased vein wall stiffness during the resolution process [164]. Similarly, collagenolysis seems to occur within the first seven days of thrombus formation in a murine model [165]. MMP-2 and MMP-9 are suggested to be the most important enzymes in this process as they not only have protein degradative properties, but also facilitate monocyte invasion, neovascularization and regulate plasminogen activation, as well as a number of growth factor pathways [166,167,168,169,170]. Deletion of MMP-9 is associated with decreased vein wall fibrosis in experimental stasis DVT, likely directed by monocytes [171]. Similarly, deletion of the CCR2 receptor impairs thrombus resolution, associated with a reduction in MMP-2 and MMP-9 [172].

There is extensive evidence to suggest that ROS modulate both MMP gene expression and MMP activation [173]. Extracellular ROS were shown to increase expression of various MMPs in vitro, including MMP-2 and MMP-9 in endothelial cells and macrophage-derived foam cells [174,175,176], with ROS derived from NOX enzymes, xanthine oxidase and mitochondria responsible for MMP-1 and MMP-9 generation [177,178]. Pharmacological antioxidants [178,179] or genetically overexpressed antioxidant enzymes [180] have the opposite effect. Intracellular signaling pathways important for MMPs and their endogenous inhibitors (TIMPs), such as Ras and MAPK, are also modulated by ROS through oxidative phosphatase inactivation [181]. Integrin ligand-binding is one of the most important activation mechanisms of MMPs and is dependent on ROS derived from NOX enzymes, 5-lipoxygenase and mitochondrial release [181].

## 15. Diseases with Increased Risk of DVT and Involving ROS

A number of conditions increase the risk for DVT and the mechanisms regulating many of these have been identified to involve dysregulated ROS levels. An example of this is Antiphospholipid Syndrome (APS). Venous and arterial thrombi are major manifestations of this condition and endosomal redox signaling has been identified as an important pathophysiological mechanism driving thrombus formation [182]. The formation of venous thrombi is inhibited, for example, in a mouse NOX2 knockout APS model [183]. APS patients with thrombosis also carry higher circulating levels of reduced Factor XI (FXI), one of the constituents of the coagulation cascade [184]. This reduced form of FXI is generated by the action of oxidoreductases (TRX-1) and is more easily activated by thrombin than the oxidized form, giving a more procoagulant state in APS patients [184]. Hydroxychloroquine (HCQ), which has been used to treat patients with APS, inhibits proinflammatory signaling pathways by targeting endosomal NOX, further supporting an important role for ROS in APS [185].

Behçet’s disease is another autoimmune disorder with an increased risk for developing venous thrombosis. A recent study of 98 Behçet’s disease patients has shown that thrombus formation was promoted by oxidatively altered fibrinogen, which was associated with neutrophil activation and enhanced NOX-dependent ROS production [186]. 

Increased risk of DVT has also been reported for exposure to air pollution [187] and a recent study suggests that this might be mediated by increased oxidative stress [188]. Endothelial cells exposed to ultrafine particles show upregulated TF expression via NOX4 signaling, earlier thrombin generation and faster fibrin clot formation [188]. The implications of this finding in vivo remain to be explored.

Oxidative stress is associated with conditions that result from lifestyles common in western countries. One of these is Metabolic Syndrome, which is positively associated with the risk of venous thrombosis [189]. The oxidative stress in these patients leads to substantially elevated levels of oxidized low-density lipoproteins (ox-LDL), which is known to trigger platelet activation and be causally involved in prothrombotic mechanisms [52]. Ox-LDL binds to CD36 on platelets and induces their activation via NOX2 induction [190]. In line with this, the plasma of obese women contains elevated levels of the platelet activation markers soluble CD40L (sCD40L) and soluble P-selectin, associated with NOX2 upregulation in platelets [53]. 

Chronic stress is another lifestyle factor that is positively associated with ROS levels [191]. Chronic stress is associated with increased oxidative stress, platelet activation and DVT formation in an experimental model, an effect that was alleviated by antioxidant treatment [192]. ROS formation following chronic stress in another experimental model, synergistically increased by NOX enzymes through the renin-angiotensin system, and inflammatory reactions in white adipose tissue, resulted in a prothrombotic state [193]. 

DVT rates increase with age and this is thought to involve oxidative stress as aging-related venous thrombosis in mice is promoted by the redox sensor, mTORC1, via the elevation of platelet reactivity, an effect that is reduced by antioxidant therapy with NAC [194]. NAC had no beneficial effect in young mice, however, suggesting that antioxidant treatment is only effective when levels of oxidative stress are high (as is present in older mice), while the lower oxidative stress and smaller thrombus size present in younger mice is not amenable to significant treatment effects [194].

Age-related prothrombotic effects also involve the down-regulation of the protective NO pathway and upregulation of the NOX- and COXs-dependent oxidative stress pathway, promoting TF expression and activity in endothelial cells [8]. Age-dependent increased susceptibility to venous thrombosis is rescued by overexpression of the antioxidant enzyme GPX-1 [67].

## 16. Dietary and Pharmacological Antioxidants 

### 16.1. Dietary

Various nutritional factors and drugs are known to have antioxidant functions. While evidence exists that they influence various distinct processes important for DVT formation, their overall effect on DVT has yet to be established. The antioxidant effects of vitamins A, C and E for instance are well known [18]. Vitamin E reduces cardiovascular events in subgroups of patients with increased oxidative stress [195], but supplementation in the general population holds no cardiovascular benefit, and may even increase all-cause mortality [196]. Data on how vitamin E might influence the formation and/or resolution of DVT are sparse and limited to two components of the DVT formation process, platelet activation and the coagulation cascade. Activation of isolated platelets with collagen can be inhibited by the antioxidative effects of vitamin E [65]. Vitamin E downregulates the in vitro expression of the initiating protein of the coagulation cascade, TF, in monocytes, suggestive of an anticoagulant function [197,198]. 

In hemodialysis, the extracorporeal circuit leads to activation of the clotting cascade, which is associated with increased ROS generation and reduced antioxidant mechanisms [199]. The use of a less thrombogenic ethylene–vinyl–alcohol (EVAL) dialysis membrane inhibits NOX2-mediated ROS production, and the use of other materials with ROS-scavenging activities results in the reduced activation of clotting [200,201]. The administration of natural antioxidants, such as red grape juice, which has antioxidant properties, reduces neutrophil NOX activity and plasma concentrations of oxidized LDL (ox-LDL) to an even greater extent than vitamin E in patients undergoing hemodialysis [199]. 

Beer contains a nutritional antioxidant, xanthohumol, for which a direct beneficial effect for the development of DVT has been shown. Xanthohumol, present in hops that are used in beer making, prevents both arterial and venous thrombosis in mice by decreasing ROS accumulation and inhibiting platelet activation without increased bleeding risk [202]. Moderate beer consumption is associated with a decreased risk of venous thrombosis [202,203,204]. Red wine inhibits platelet activation and diminishes experimental venous thrombosis in rats, an effect that was associated with a substantial increase in total radical-trapping antioxidant parameters (TRAP) [205]. Antioxidant polyphenols, present in a variety of foods and drinks, including red wine and chocolate, are known to reduce platelet reactivity [206] and this is believed to be one of the major drivers of improved cardiovascular mortality when wine is moderately consumed [207]. The Mediterranean diet, and olive oil in particular, also favor antioxidant mechanisms. A cross-over study comparing olive oil with corn oil revealed olive oil intake caused a reduced post-prandial ROS increase in serum and platelets via NOX2 downregulation [208]. It appears, therefore, that nutritional antioxidants can modulate several important steps in the DVT formation process, but their overall benefit in reducing this condition remains to be established.

### 16.2. Pharmaceutical

The beneficial effects of statins in cardiovascular disease are mainly attributed to their lipid lowering effects, but there is extensive evidence to suggest that they have pleiotropic effects in regulating disease. Statins decrease oxidative stress and platelet activation in hypercholesterolemic patients by an early and late mechanism. The early effect is dependent on the direct inhibition of platelet NOX2 and independent of any lipid lowering effect, while the late effect is associated with LDL lowering including the platelet-activating molecule, ox-LDL [209,210]. These antiplatelet effects could be responsible for the reduction in thrombotic events observed upon pre-procedural statin therapy [211]. The resolution in venous thrombi is also enhanced by statin therapy, although the mechanism has yet to be reported [212]. Antiplatelets (aspirin) and anticoagulants (Xa inhibitor, rivaroxaban) also have antioxidant properties, as they reduce NOX2-mediated platelet ROS production [213,214].

Most of these drugs have already been evaluated for their benefit in DVT patients, but there are also other antioxidant drugs that are used in other fields of medicine. Modified versions and dosages of these drugs could be repurposed for use in thrombotic diseases. Thioredoxin (TRX) inhibitors, for example, are currently under investigation in clinical trials for diseases involving an imbalance in the NADPH/thioredoxin reductase/thioredoxin system, such as cancer. TRX inhibitors also attenuate platelet function and thrombus formation, which could lead to their being repurposed as antiplatelet agents [215]. The oral anti-diabetic drug, Alogliptin, diminishes oxidative stress and the associated prothrombotic state in a mouse model of chronic stress [193]. 

Drugs with pro-oxidant properties, such as the anticancer, estrogen-receptor-blocker tamoxifen, might negatively impact processes related to DVT. Platelet activation through tamoxifen-mediated NOX upregulation has previously been suggested to be causally involved in the increased risk of venous thrombosis in breast cancer patients treated with tamoxifen [216].

## 17. Conclusions

ROS are involved in the regulation of all of the major processes that promote the formation of venous thrombi. These include coagulation; platelet reactivity; and sterile inflammation (for example NETosis) during formation. Oxidative stress also appears to favor the ‘intravascular wound healing’ processes that control the remodeling of a venous thrombus and adjacent vein wall including fibrinolysis; sterile inflammation (monocyte accumulation); extracellular matrix deposition and its remodeling; and neovascularization. 

Dysregulated redox control is a common feature of conditions with an elevated risk of venous thrombosis, and therefore restoring oxidative balance might represent a possible therapeutic option in some of these patients. However, the ubiquitous deployment of ROS in the regulation of both physiological and pathophysiological redox regulatory mechanisms poses challenges in determining which redox pathways can be targeted for therapeutic benefit without affecting normal hemostasis.

## Abbreviations

AP-1	Activator protein 1
APC	Activated protein C
APS	Antiphospholipid Syndrome
ATIII	Antithrombin III
AQP	Aquaporin channel
CAM	Cell adhesion molecule
CCR2	C-C chemokine receptor type 2
CGD	X-linked chronic granulomatous disease
COX	Cyclooxygenase
CVD	Cardiovascular disease
CAM	Cell adhesion molecule
CTEPH	Chronic thromboembolic pulmonary hypertension
DVT	Deep vein thrombosis
ECM	Extracellular matrix
EVAL	Ethylene-vinyl-alcohol
fMLP	N-formyl-met-leu-phe
FX	Factor X
FXI	Factor XI
GPVI	Glycoprotein VI
GPX	Glutathione peroxidase
Hb	Hemoglobin
HCQ	Hydroxychloroquine
HIF	Hypoxia-inducible factor
HO	Heme oxygenase
HUVEC	Human umbilical vein endothelial cell
IgE	Immunoglobulin E
IL-8	Interleukin-8
IVC	Inferior vena cava
LPS	Lipopolysaccharide
MAPK	Mitogen-activated protein kinase
MC	Mast cell
MCP-1	Monocyte chemotactic protein 1
MMP	Matrix metalloproteinase
MPO	Myeloperoxidase
mTORC1	Mammalian target of rapamycin complex 1
MyD88	Myeloid differentiation primary response 88
NAC	N-acetylcysteine
NF-κB	Nuclear factor kappa-light-chain-enhancer of activated B cells
NLRP3	NLR family pyrin domain containing 3
NO	Nitric oxide
NOX	Nicotinamide adenine dinucleotide phosphate (NADPH) oxidase
Nrf2	Nuclear factor erythroid 2-related factor 2
NE	Neutrophil elastase
NET	Neutrophil extracellular trap
Orai1	Calcium release-activated calcium channel protein 1
Ox-LDL	Oxidized low-density lipoprotein
PA	Pulmonary artery
PAD4	Protein-arginine deiminase type-4
PAI	Plasminogen activator inhibitor
PE	Pulmonary embolism
PECAM-1	Platelet endothelial cell adhesion molecule 1
PMA	Phorbol myristate acetate
PSGL-1	P-selectin glycoprotein ligand-1
IVC	Inferior vena cava
ROS	Reactive oxygen species
SOCE	Store-operated calcium entry
STIM1	Stromal interaction molecule 1
TF	Tissue factor
TFPI	Tissue factor pathway inhibitor
TGF-β	Transforming growth factor beta
TIMP	Tissue inhibitor of metalloproteinases
TLR	Toll-like receptor
TM	Thrombomodulin
TNF-α	Tumor necrosis factor alpha
tPA	Tissue-type plasminogen activator
TRPC6	Transient receptor potential channel 6
TRX	Thioredoxin
uPA	Urokinase-type plasminogen activator
VEGF	Vascular endothelial growth factor
VSMC	Vascular smooth muscle cells
VTE	Venous thromboembolism
ZPI	Protein Z-dependent protease inhibitor
